# A Gaussian Process Model for Color Camera Characterization: Assessment in Outdoor Levantine Rock Art Scenes

**DOI:** 10.3390/s19214610

**Published:** 2019-10-23

**Authors:** Adolfo Molada-Tebar, Gabriel Riutort-Mayol, Ángel Marqués-Mateu, José Luis Lerma

**Affiliations:** Department of Cartographic Engineering, Geodesy, and Photogrammetry, Universitat Politècnica de València, 46022 València, Spain; gabriuma@upv.es (G.R.-M.); amarques@cgf.upv.es (Á.M.-M.); jllerma@cgf.upv.es (J.L.L.)

**Keywords:** cultural heritage, camera characterization, polynomial regression, Gaussian processes, colorimetry, CIE color spaces, noise analysis

## Abstract

In this paper, we propose a novel approach to undertake the colorimetric camera characterization procedure based on a Gaussian process (GP). GPs are powerful and flexible nonparametric models for multivariate nonlinear functions. To validate the GP model, we compare the results achieved with a second-order polynomial model, which is the most widely used regression model for characterization purposes. We applied the methodology on a set of raw images of rock art scenes collected with two different Single Lens Reflex (SLR) cameras. A leave-one-out cross-validation (LOOCV) procedure was used to assess the predictive performance of the models in terms of CIE XYZ residuals and $\Delta E_{ab}^{*}$ color differences. Values of less than 3 CIELAB units were achieved for $\Delta E_{ab}^{*}$. The output sRGB characterized images show that both regression models are suitable for practical applications in cultural heritage documentation. However, the results show that colorimetric characterization based on the Gaussian process provides significantly better results, with lower values for residuals and $\Delta E_{ab}^{*}$. We also analyzed the induced noise into the output image after applying the camera characterization. As the noise depends on the specific camera, proper camera selection is essential for the photogrammetric work.

## 1. Introduction

Accurate recording of color is one of the fundamental tasks in many scientific disciplines, such as chemistry, industry, medicine, or geosciences to name just a few. Color measurement is a crucial aspect in archaeology and specifically in rock art documentation [1,2]. The correct measurement of color allows researchers to study, diagnose, and describe rock art specimens and detect chromatic changes or alterations over time. High-precision metric models together with reliable color information data sets provide essential information in modern conservation and preservation works.

The appropriate description of color is not a trivial issue in cultural heritage documentation [3,4]. Color is a matter of perception, which largely depends on the subjectivity of the observer. Therefore, correct color registration requires objective colorimetric measurement described in rigorous color spaces. Usually, color spaces defined by the CIE are used as the standard reference framework for colorimetric measurement and management.

To avoid damage into the pigment, direct contact measurements with colorimeters or spectrophotometers on painted rock art panels are not allowed. Instead, indirect and noninvasive methods for color determination are required. Thus, the use of digitization techniques with conventional digital cameras to support rock art documentation is becoming more and more frequent [5,6,7,8].

The color information obtained from digital images can be easily captured, stored, and processed. The drawback of such color information lays on the response recorded by the sensor, which is not strictly colorimetric. The RGB responses do not satisfy the Luther–Ives condition so that RGB data are not a linear combination of CIE XYZ coordinates [9]. If the values recorded in the RGB channels were proportional to the input energy, a simple linear relationship between the RGB data acquired by the digital camera and the CIE XYZ tristimulus values would exist. However, in general, the spectral sensitivities of the three RGB channels are not linear combinations of the color-matching functions [10]. The signals generated by digital cameras are indeed referred to as “device dependent”. Thus, a transformation to convert the input RGB data into device-independent color spaces is necessary.

A widely accepted approach to establish the mathematical relationships between the original RGB data and well-defined independent color spaces is the procedure of digital camera characterization [10]. Different techniques are used for colorimetric camera characterization. Numerous papers have been written regarding common techniques, such as polynomial transformation with least-squares regressions [9,10], interpolation from look-up tables [11], artificial neural networks [12], and principal component analysis [13]. Further studies have focused on optimizing characterization, including the use of pattern search optimization [14], multiple regression [15], root-polynomial regression [16], or spectral reflectance reconstruction [17,18,19].

The colorimetric characterization of digital cameras based on polynomial models is an appropriate starting point; they are widely accepted, mathematically simpler and require smaller training sets and less computing time [20,21]. Previous experiments using second-order polynomials applied in rock art paintings gave also good results [22]. However, they tend to be rigid models and suffer from overestimation or underestimation when many or few data are provided. Furthermore, it is known the lack of reliable generalization of predictions in polynomial models, especially when extrapolating or in the case of modeling wiggly functions [23]. Therefore, it is desirable to improve the results by means of flexible, robust and more accurate models.

In this work, we introduce a novel approach for documenting rock art paintings based on a Gaussian process (GP) model. GPs are natural, flexible nonparametric models for *N*-dimensional functions, with multivariate predictors (input variables) in each dimension [24,25]. The defining property of a GP model is that any finite set of values is jointly distributed as a multivariate Gaussian function. A GP is completely defined by its mean and covariance function. The covariance function is the crucial ingredient in a Gaussian process as it encodes correlation structure which characterizes the relationships between function values at different inputs. GP allows not only nonlinear effects and handling implicit interactions between covariates, but also improves generalization of function values for both interpolation and extrapolation purposes. Due to their generality and flexibility, GPs are of broad interest across machine learning and statistics [25,26].

GP models are formulated and estimated within a Bayesian framework, and all inference is based on the multivariate posterior distribution. Computing the posterior distribution is often difficult, and for this reason, different computation approaches can be used. The Markov chain Monte Carlo (MCMC) is a sampling method that provides a sample of the joint posterior distribution of the parameters [27,28].

The GP model results were compared to the common approach based on polynomial regression models. The main advantage of nonparametric over parametric models is their flexibility [29,30]. In a parametric framework, the shape of the functional relationship is a prespecified, either linear or nonlinear, function, limiting the flexibility of the modeling. In a nonparametric framework, the shape of the functional relationship is completely determined by the data, allowing for a higher degree of adaptability.

The goodness of fit and predictive performance of the models are assessed by analyzing the adjustment residuals and the leave-one-out cross-validation (LOOCV) residuals. The quality of the characterized image is also evaluated in terms of colorimetric accuracy by means of color differences among observed and fitted colors using the CIE framework. In addition, we evaluate the induced noise into the output image after the characterization which is recognized as a drawback for some image applications such as image matching or pattern recognition. The induced noise is evaluated by computing and comparing the coefficients of variation of digital values between the original and output images.

## 2. Materials and Methods

### 2.1. Case Study: Cova dels Cavalls

The working area is a rock art scene located in Shelter II of the Cova dels Cavalls site in the county of Tirig, province of Castelló (Spain). This cave is part of one of the most singular rock art sites of the Mediterranean Basin in the Iberian Peninsula, which is listed by UNESCO as a World Heritage since 1998 [31].

The images were taken using two different SLR digital cameras, a Sigma SD15 and a Fujifilm IS PRO. The images contain the hunting scene located in the central part of this emblematic archaeological site. Parameters such as focal, exposure time, aperture, and ISO were controlled during the photographic sessions for both cameras. Photographs were taken in the raw format under homogeneous illumination conditions.

The main difference between the Fujifilm IS PRO and the Sigma SD15 cameras is their integrated sensors. The Fujifilm incorporates a 12 megapixels Super CCD imaging sensor, with resolution of 4256 × 2848 pixels and a color filter array (CFA) with a Bayer pattern. The use of CFA implies that the color registered in every individual pixel is not acquired directly but as a result of interpolation between channels. On the other hand, the Sigma carries a three-layer CMOS Foveon^®^X3 sensor of 2640 × 1760 pixels, which makes it a true trichromatic digital camera [32]. The main advantage of this sensor is its ability to capture color without any interpolation.

### 2.2. Image-Based Camera Characterization Methodology

**Figure 1:**
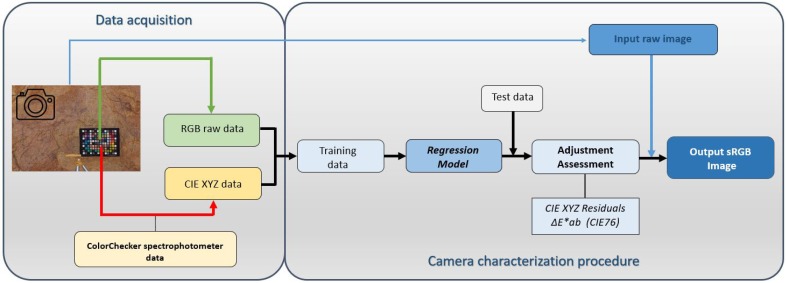
Schematic diagram designed for the camera characterization.

The output RGB digital values registered by the camera depend on three main factors: the sensor architecture, the lighting conditions, and the object being imaged. Even assuming the same object and lighting conditions, other factors can still produce different RGB responses within and across scenes. Some elements such as the internal color filters or user settings (exposure time, aperture, white balance, and so on) can modify the output digital values. As a result, the original RGB data registered by the sensor cannot be used rigorously for the quantitative determination of color, and native RGB camera color spaces are said to be device dependent. A way to transform the signal captured by the camera sensor into a physicaly-based, device-independent color space is by means of camera characterization (See workflow in Figure 1).

To carry out the characterization, various training and test datasets are required. An important aspect on the camera characterization process is the establishment of the working color spaces. Some of the most common color spaces used are the input RGB data and the output tristimulus coordinates [10]. In the preliminary stages of the study four different transformations, between color spaces were tested, including RGB–CIE XYZ, RGB-CIELAB, LMS–CIE XYZ, and LMS–CIELAB. The transformation that worked the best was the RGB–CIE XYZ, whose results are reported in the rest of the paper.

**Figure 2:**
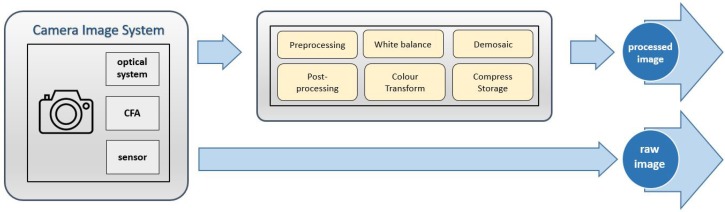
Raw images versus processed images workflow.

On the other hand, the digital RGB values are available after a complex process driven by the built-in software and electronics of the camera [33]. Usually, a set of preprocessing operations, e.g., demosaicing, white balance, gamut mapping, color enhancement, or compression, are applied automatically to the raw image (Figure 2). It is thus preferable to work with raw data versus RGB processed or compressed image files.

The raw RGB training and test sample data were extracted from the images using our software pyColourimetry which was developed in previous research. This software was written in the Python programming language following the CIE recommendations. It allows raw RGB data extraction from conventional camera formats and implements other colorimetric functions such as transformation among color spaces, color difference calculation, or spectral data treatment [34].

Also, the data acquisition includes the direct measurement of the tristimulus values of the patches present in the color chart and the raw RGB data extraction from the digital image. Thus, a color chart has to be included as a colorimetric reference in the photographic shot to carry out the camera characterization. For this experiment, we used an X-Rite ColorChecker SG Digital Color Chart as a color standard. This chart is routinely used in digital photography for color calibration. It consists of an array of 140 color patches. The number of patches is supposedly enough to cover the color domain present in the scene as well as to provide training and test data sets to analyze the results after the camera characterization.

CIE XYZ values for the ColorChecker patches have to be known prior to undertake the camera characterization. An accepted option is to use those tristimulus values provided by the manufacturer. Nevertheless, it is highly recommended to carry out a new measurement, preferably by means of a colorimeter or spectrophotometer, using the setup of the specific experiment. The spectral reflectance data were acquired using the spectrophotometer Konica Minolta CM-600d, following CIE recommendations (2° standard observer under D65 illuminant). CIE XYZ coordinates can be obtained by transforming the spectral data using well-known CIE formulae [35].

To visualize the tristimulus coordinates, it is necessary to perform a final transformation of the CIE XYZ values into the sRGB color space, which is compatible with most digital devices. This transformation is carried out based on the technical recommendations published by the International Electrotechnical Commission [36]. Thus, the final outcome of the characterization consists of an sRGB output image for each regression model.

Once the digital camera is colorimetrically characterized, it can be used for the rigorous measurement of color simulating a colorimeter [37]. Using a characterized camera, we can obtain accurate color information over complete scenes, which is a very important requirement to properly analyze rock art. The use of conventional cameras for color measurement allows researchers to take pictures with low-cost recording devices, suitable for carrying out heritage documentation tasks using noninvasive methodologies [22].

### 2.3. Gaussian Processes for Camera Characterization

The main aim of camera characterization is to find the mapping function between the RGB color values and the CIE XYZ tristimulus coordinates:

(1) $$f:RGB\in {I\;R}^{3}\to XYZ\in {I\;R}^{3}$$

Commonly, this multivariate mapping function is divided into independent functions for each each single XYZ tristimulus value. In this paper, we propose a Gaussian process (GP) to estimate these functions, with different model parameters, $\theta_{1}$, $\theta_{2}$, and $\theta_{3}$, for each mapping function:

$$f_{1}:RGB\in {I\;R}^{3}\overset{GP(\theta_{1})}{\to }X\in I\;R$$

(2) $$f_{2}:RGB\in {I\;R}^{3}\overset{GP(\theta_{2})}{\to }Y\in I\;R$$

$$f_{3}:RGB\in {I\;R}^{3}\overset{GP(\theta_{3})}{\to }Z\in I\;R$$

#### 2.3.1. Gaussian Process Model

A GP is a stochastic process which defines the distribution over a collection of random variables [24,25]. The defining property of a GP is that any finite set of random variables is jointly distributed as a multivariate normal distribution. A GP is completely characterized by its mean and covariance functions that control the a priori behavior of the function. GP can be used as prior probability distributions for latent functions in generalized linear models [38]. However, in this paper, we focus on GP in linear models (a normal outcome), as we can assume that the CIE XYZ color coordinates are normally distributed.

A GP for a normal outcome $y=\left\{y_{1},y_{2},\cdots ,y_{n}\in I\;R\right\}\in {I\;R}^{n}$, paired with a matrix of *D* inputs variables (predictors) $X=\left\{x_{1},x_{2},\cdots ,x_{3}\in {I\;R}^{n}\right\}\in {I\;R}^{n\times D}$, consists of defining a multivariate Gaussian distribution over y conditioned on X:

(3) $$y|X∼N(\mu \left(X\right),K\left(X|\theta \right)+\sigma^{2}I)$$

where μ(X) is a n-vector, K(X|θ) is an n×n covariance matrix, $\sigma^{2}$ is the noise variance, and *I* the n×n diagonal identity matrix. The mean function $\mu :X\in {I\;R}^{n\times D}\to {I\;R}^{n}$ can be anything, although it is usually recommended to be a linear model or even zero. The covariance function $K|\theta :X\in {I\;R}^{n\times D}\to {I\;R}^{n\times n}$ must be a positive semidefinite matrix [25,26]. In this work, we use the square exponential covariance function, which is the most commonly used function of the Matérn class of isotropic covariance functions [25]. The squared exponential covariance function for two observed points *i* and *j* (i,j=1,⋯,n) takes the form

(4) $$K{\left(X,\theta \right)}_{ij}=\alpha^{2}\exp\left(-\frac{1}{2}\sum_{d=1}^{D}\frac{1}{\ell_{d}^{2}}{\left(x_{di}-x_{dj}\right)}^{2}\right)$$

where θ={α,ℓ}; α is the marginal variance parameter, which controls the overall scale or magnitude of the range of values of the GP; and $\ell ={\left\{\ell_{d}\right\}}_{d=1}^{D}$ are the lengthscale parameters, which control the smoothness of the function in the direction of the *d*-predictor, so that the larger the lengthscale the smoother the function.

#### 2.3.2. Bayesian Inference

Bayesian inference is based on the joint posterior distribution $p(\theta ,\sigma^{2}|y,X)$ of parameters given the data, which is proportional to the product of the likelihood and prior distributions,

(5) $$p\left(\theta ,\sigma^{2}|y,X\right)\propto p\left(y|\theta ,\sigma^{2},X\right)p\left(\theta \right)p\left(\sigma^{2}\right)$$

In the previous equation,

$$p\left(y|\theta ,\sigma^{2},X\right)=\prod_{i}N\left(y_{i}|0,K_{ii}\left(X|\theta \right)+\sigma^{2}\right)$$

is the likelihood of the model, where the mean function μ(X) has been set to zero for the sake of simplicity, and

$$\begin{array}{cc} p(\alpha ) & =N(\alpha |0,10)\\ p(\ell ) & =\prod_{d=1}^{D}\mathrm{Gamma}\left(\ell_{d}|1,0.1\right)\\ p(\sigma^{2}) & =N^{+}\left(\sigma^{2}|0,10\right\} \end{array}$$

are the prior distributions of the parameters of the model. These correspond to weakly informative prior distributions based on prior knowledge about the magnitude of the parameters.

Predictive inference for new function values $\tilde{y}$ for a new sequence of input values $\tilde{X}$ can be computed by integrating over the joint posterior distribution

(6) $$p\left(\tilde{y}|y\right)=\int p\left(\tilde{y}|\theta ,\sigma^{2},\tilde{X}\right)p\left(\theta ,\sigma^{2}|y,X\right)d\theta d\sigma^{2}$$

To estimate both the parameter posterior distribution and the posterior predictive distribution for this model, simulation methods and/or distributional approximations methods [38] must be used. Simulation methods based on MCMC [27] are general sampling methods to obtain samples from the joint posterior distribution. For quick inferences and large data sets, where iterative simulation algorithms are too slow, modal and distributional approximation methods can be more efficient and approximate alternatives.

### 2.4. Second-Order Polynomial MOdel

This is the most extended model in practical camera characterization. The *N*-dimensional collections of random observations X, Y, and Z are the CIE color variables, where $X_{i}$, $Y_{i}$, and $Z_{i}$ represent the color coordinates of the *i*th order observation *i* (i=1,⋯,N). Each X, Y, and Z *N*-dimensional variable is considered to follow a normal distribution depending on an underlying second-order polynomial function f and noise variance $\sigma^{2}$,

$$p\left(X|f_{x},\sigma_{x}\right)=N\left(X|f_{x},\sigma_{x}^{2}I\right)$$

(7) $$p\left(Y|f_{y},\sigma_{y}\right)=N\left(Y|f_{y},\sigma_{y}^{2}I\right)$$

$$p\left(Z|f_{z},\sigma_{z}\right)=N\left(Z|f_{z},\sigma_{z}^{2}I\right)$$

where *I* is the N×N identity matrix. The latent second-order polynomials functions $f_{x}$, $f_{y}$, and $f_{z}$ take the form

$$f_{x}=a_{0}+a_{1}\;\cdot \;R+a_{2}\;\cdot \;G+a_{3}\;\cdot \;B+a_{4}\;\cdot \;R\;\cdot \;G+a_{5}\;\cdot \;R\;\cdot \;B+a_{6}\;\cdot \;G\;\cdot \;B+a_{7}\;\cdot \;R^{2}+a_{8}\;\cdot \;G^{2}+a_{9}\;\cdot \;B^{2}$$

(8) $$f_{y}=b_{0}+b_{1}\;\cdot \;R+b_{2}\;\cdot \;G+b_{3}\;\cdot \;B+b_{4}\;\cdot \;R\;\cdot \;G+b_{5}\;\cdot \;R\;\cdot \;B+b_{6}\;\cdot \;G\;\cdot \;B+b_{7}\;\cdot \;R^{2}+b_{8}\;\cdot \;G^{2}+b_{9}\;\cdot \;B^{2}$$

$$f_{z}=c_{0}+c_{1}\;\cdot \;R+c_{2}\;\cdot \;G+c_{3}\;\cdot \;B+c_{4}\;\cdot \;R\;\cdot \;G+c_{5}\;\cdot \;R\;\cdot \;B+c_{6}\;\cdot \;G\;\cdot \;B+c_{7}\;\cdot \;R^{2}+c_{8}\;\cdot \;G^{2}+c_{9}\;\cdot \;B^{2}$$

where the vectors $a=a_{1},\cdots ,a_{9}$, $b=b_{1},\cdots ,b_{9}$ and $c=c_{1},\cdots ,c_{9}$ represent the polynomial coefficients, and R, G, and B are the *N*-dimensional variables in the input RGB space.

The likelihood functions of the variables X, Y and Z (given the coefficients a, b, c), the variance parameters $\sigma^{2}=\sigma_{x}^{2},\sigma_{y}^{2},\sigma_{z}^{2}$, and the variables R, G and B, take the form

$$p\left(X|a,\sigma_{x},R,G,B\right)=\prod_{i}^{N}N\left(X_{i}|a,\sigma_{x}^{2},R_{i},G_{i},B_{i}\right)$$

(9) $$p\left(Y|b,\sigma_{y},R,G,B\right)=\prod_{i}^{N}N\left(Y_{i}|b,\sigma_{y}^{2},R_{i},G_{i},B_{i}\right)$$

$$p\left(Z|c,\sigma_{z},R,G,B\right)=\prod_{i}^{N}N\left(Z_{i}|c,\sigma_{z}^{2},R_{i},G_{i},B_{i}\right)$$

where the subscript *i* represents the *i*th observed value.

#### Bayesian Inference

The joint posterior distributions are proportional to the product of the likelihood and prior distributions:

$$\begin{array}{c} p\left(a,\sigma_{x}^{2}|X\right)\propto p\left(X|a,\sigma_{x}^{2},R,G,B\right)p\left(a\right)p\left(\sigma_{x}^{2}\right)\\ p\left(b,\sigma_{y}^{2}|Y\right)\propto p\left(Y|b,\sigma_{y}^{2},R,G,B\right)p\left(b\right)p\left(\sigma_{y}^{2}\right)\\ p\left(c,\sigma_{z}^{2}|Z\right)\propto p\left(Z|c,\sigma_{z}^{2},R,G,B\right)p\left(c\right)p\left(\sigma_{z}^{2}\right) \end{array}$$

We set vague prior distributions p(a)=N(a|0,1000), p(b)=N(b|0,1000), p(c)=N(c|0,1000), and $p\left(\sigma \right)=N^{+}\left(\sigma |0,1\right)$ for the hyperparameters a, b, c, and σ, respectively, based on prior knowledge about the magnitude of the parameters.

Predictive inference for new function values $\tilde{X},\tilde{Y},$ and $\tilde{Z}$ for a new sequence of input values $\tilde{R},\tilde{G},$ and $\tilde{B}$ can be computed by integrating over the joint posterior distributions

$$\begin{array}{c} p\left(\tilde{X}|X\right)=\int p\left(\tilde{X}|a,\sigma_{x}^{2},\tilde{R},\tilde{G},\tilde{B}\right)p\left(a,\sigma_{x}^{2}|X\right)dad\sigma_{x}^{2}\\ p\left(\tilde{Y}|Y\right)=\int p\left(\tilde{Y}|b,\sigma_{y}^{2},\tilde{R},\tilde{G},\tilde{B}\right)p\left(b,\sigma_{y}^{2}|Y\right)dbd\sigma_{y}^{2}\\ p\left(\tilde{Z}|Z\right)=\int p\left(\tilde{Z}|c,\sigma_{z}^{2},\tilde{R},\tilde{G},\tilde{B}\right)p\left(c,\sigma_{z}^{2}|Z\right)dcd\sigma_{z}^{2} \end{array}$$

Simulation methods based on MCMC are used for estimating both the parameter posterior distribution and the posterior predictive distribution of these models.

### 2.5. Model Checking and Comparison

For model assessment, common checking procedures of normality, magnitude and tendencies on the fitted and predicted residuals are used. Fitted residuals can be useful for identifying outliers or misspecified models and give us the goodness of the fit for the sampling patches. Furthermore, the performance of each model was assessed using the LOOCV approach [39]. The LOOCV procedure has been previously used in color science multiple times [40,41,42,43], although its origins can be traced back to early practical statistics methods [39] and is routinely used in modern data science applications [44].

In our study, the LOOCV consists of setting aside an individual patch and calculating the prediction model. Then, the predicted value is compared to its observed value which gives a measure of the model predictive accuracy. This allows obtaining an average of the predictive accuracy for unobserved patches as well as individual quality indicators for each color patch.

In addition to the residual analysis, it is required the assessment of the models using colorimetry metrics [1]. Also, a LOOCV procedure was conducted to assess the predictive performance in terms of color differences. In classical colorimetry, color difference metrics are determined using formulas based on the CIELAB color space, such as $\Delta E_{ab}^{*}$, also known as the CIE76 color difference [35].

The CIE XYZ color space is not uniform, that is, equal distances in this space do not represent equally perceptible differences between color stimuli. Contrarily, CIELAB coordinates are nonlinear functions of CIE XYZ, and more perceptually uniform than the CIE XYZ color space [35,45]. The $\Delta E_{ab}^{*}$ between the theoretical tristimulus coordinates against the predicted values are computed, which gives a measure of the model adjustment in a well-defined color metric.

Other modern color difference formulas which take $\Delta E_{ab}^{*}$ as a reference have been developed by the CIE. An example is the CIEDE2000 formula, which includes corrections for variations in color difference perception due to lightness, chroma, hue, and chroma–hue interaction [46,47,48]. It must be indicated that CIEDE2000 was designed for specialized industry applications [49]. To use the CIEDE2000 formula, a number of specific requirements have to be fulfilled. Some of these requirements are the sample size (greater than 4 degrees), sample–sensor separation (contact), background field (uniform, neutral gray), and sample homogeneity (textureless). Usually, these conditions cannot be guaranteed in the usual working environments found in rock art documentation. Therefore, it seems more appropriate to use the CIE76 formula herein instead of the CIEDE2000 to determine color differences.

$\Delta E_{ab}^{*}$ is calculated as the Euclidean distance between two color stimuli in CIELAB coordinates

(10) $$\Delta E_{ab}^{*}=\sqrt{{\left(\Delta L^{*}\right)}^{2}+{\left(\Delta a^{*}\right)}^{2}+{\left(\Delta b^{*}\right)}^{2}}$$

where $\Delta L^{*}$, $\Delta a^{*}$, and $\Delta b^{*}$ are the differences between the $L^{*}$, $a^{*}$, and $b^{*}$ coordinates of the two color stimuli.

Numerous guides seek to quantify the maximum value allowed (tolerance) for an acceptable color difference so that it is imperceptible by human vision. This concept is known as ”Just Noticeable Difference” (JND). A good reference is found in the Metamorfoze guideline, which employs the CIE76 color difference formula, and establishes a color accuracy of 4 CIELAB units [50].

### 2.6. Induced Noise Analysis

The radiometric response of a digital camera is the outcome of a number of factors, such as electromagnetic radiation, sensor electronics, the optical system, and so forth [51,52,53,54,55]. The noise present on a single image is basically composed of two components: the photoresponse noise of every sensor element (pixel) and the spatial nonuniformity or fixed pattern noise of the sensor array [56,57].

The nonlinear transformation functions in camera characterization models modify the input data which are themselves affected by noise. In the camera characterization, noise is transferred from the original image to the characterized image and transformed in different ways. In this paper, the analysis of noise is carried out by comparing the coefficients of variation in the original and the characterized images. The noise assessment was conducted on four selected patches of the color checker (C7, D7, C8, and D8).

## 3. Results and Discussion

### 3.1. Model Performance Assessment

For model assessment we processed the CIE XYZ residuals and the $\Delta E_{ab}^{*}$ color differences values after the characterization procedure.

#### 3.1.1. CIE XYZ Residuals

**Figure 3:**
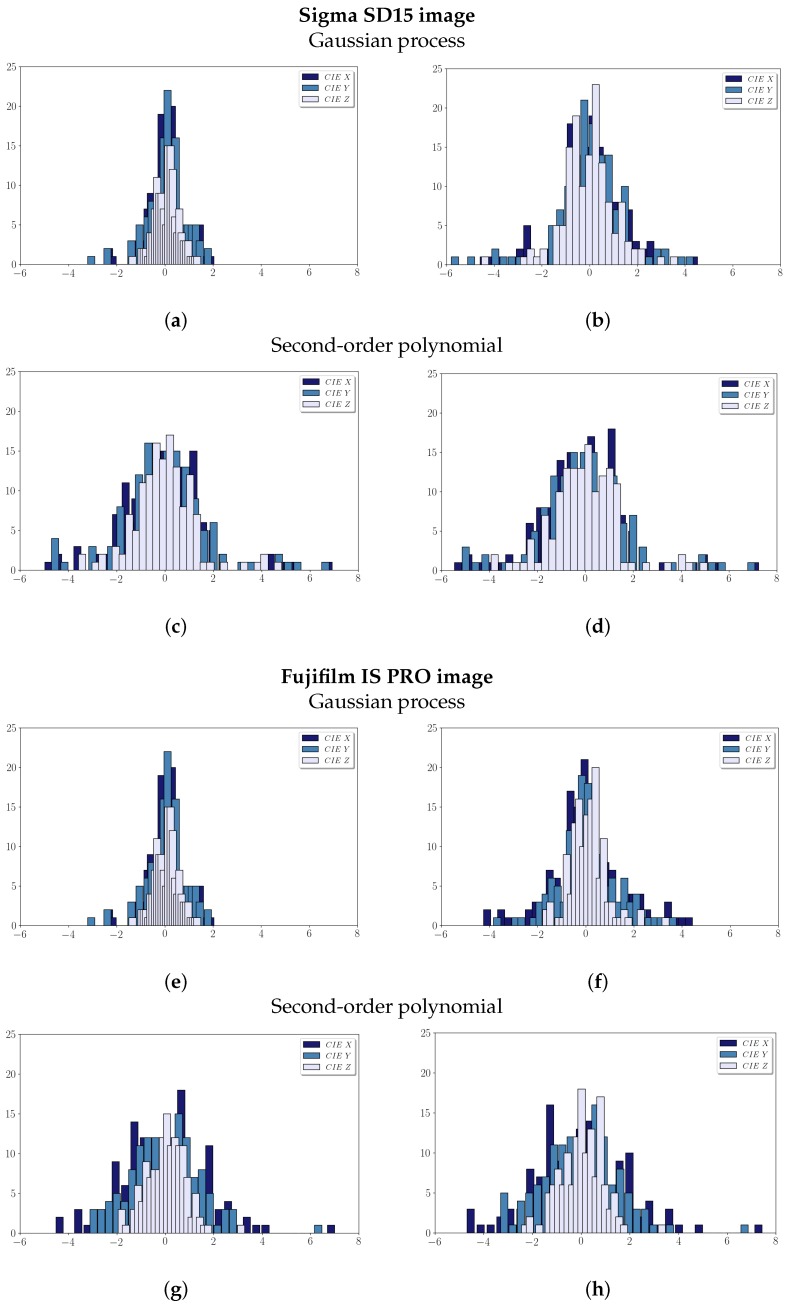
CIE XYZ residuals histograms after the adjustment: (**a**,**b**,**e**,**f**) Gaussian process; (**a**,**d**,**g**,**h**); Second-order polynomial; (**a**,**c**,**e**,**g**); CIE XYZ residuals; (**b**,**d**,**f**,**h**); LOOCV residuals.

Table 1 summarizes the fitted CIE XYZ and LOOCV residuals values achieved after the characterization process for the two cameras used in the study. Also, the histograms of the fitted and LOOCV residuals in both images are in Figure 3. Although both methodologies give satisfactory results and fit appropriately to the input RGB data, all summary statistics and histograms clearly show that GP outperforms the second-order polynomial regression model in both images. The standard deviation values, which represent the mean error, as well as the maximum and minimum residuals, are lower using the GP process than the second-order polynomial model.

Thus, given the results achieved using the GP, a notable improvement can be observed compared with the common second-order polynomial models, that is, a higher adjustment correlation coefficient and a greater predictive capacity were achieved. An improvement in the predictive capacity (LOOCV residuals) implies a better model generalization, that is, a better capacity for interpolation and extrapolation. This is a key aspect in the characterization procedure, as the output digital image is the result of the application of the model established.

#### 3.1.2. $\Delta E_{ab}^{*}$ Color Differences

**Figure 4:**
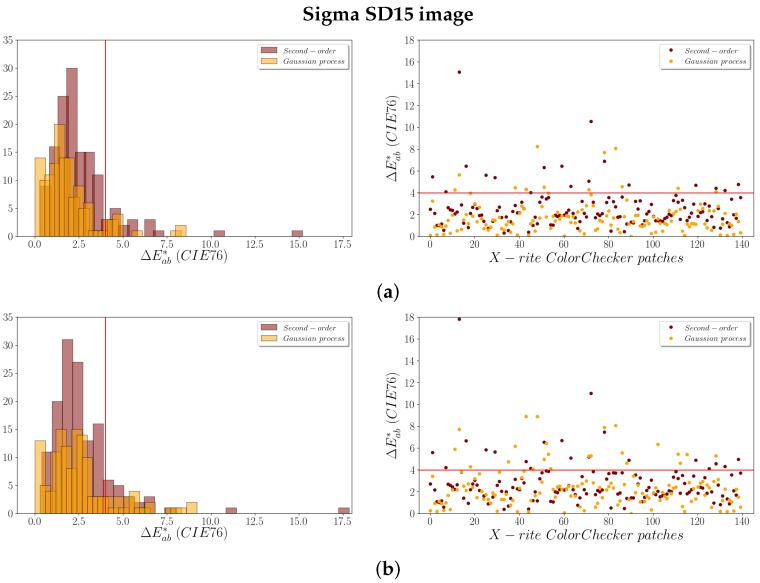
Sigma SD15 $\Delta E_{ab}^{*}$ values for the X-Rite patches: (**a**) $\Delta E_{ab}^{*}$; (**b**) LOOCV $\Delta E_{ab}^{*}$.

**Figure 5:**
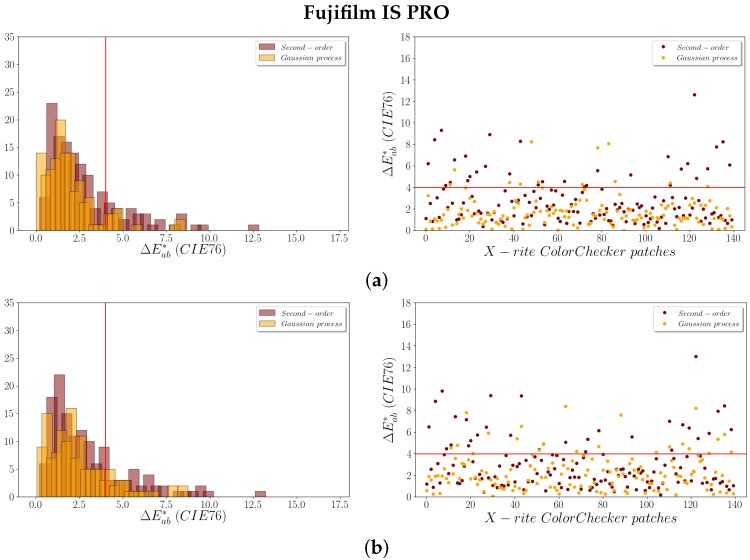
Fujifilm IS PRO $\Delta E_{ab}^{*}$ values for the X-Rite patches: (**a**) $\Delta E_{ab}^{*}$; (**b**) LOOCV $\Delta E_{ab}^{*}$.

The $\Delta E_{ab}^{*}$ color differences (Equation (10)) obtained between the theoretical and predicted values allowed us to assess the colorimetric quality achieved after the adjustment. The results obtained for the $\Delta E_{ab}^{*}$ are shown numerically in the Table 2. Also, they can be consulted graphically in the Figure 4 (for the Sigma SD15) and Figure 5 (for the Fujifilm IS PRO), where the red line delimits the JND tolerance established in 4 CIELAB units.

Under a strict colorimetric criterion, the average and median values for $\Delta E_{ab}^{*}$ obtained using both regression models are less than 3 CIELAB units, that is, lower than the JND. However, the $\Delta E_{ab}^{*}$ color differences obtained confirm that the adjustment based on the GP model offers better results than the second-order polynomial regression for both cameras. The values achieved for the mean and median $\Delta E_{ab}^{*}$ in both images are similar using the second-order polynomial regression model.

It is clearly observed that the main improvement is in the maximum $\Delta E_{ab}^{*}$ values obtained. Although the average for LOOCV $\Delta E_{ab}^{*}$ is slightly lower using the GP, the maximum values for LOOCV $\Delta E_{ab}^{*}$ decreases significantly using this model. The maximum values obtained are 17.814 and 13.021 for the Sigma SD15 and the Fujifilm IS PRO image, respectively, using a second-order polynomial regression model, whereas the maximum values for LOOCV $\Delta E_{ab}^{*}$ using the GP are ~8 CIELAB units (Table 2).

Moreover, the number of patches with $\Delta E_{ab}^{*}$ greater than 4 units (JND) clearly decrease for both images after applying the GP (Figure 4 and Figure 5). Thus, the GP improvement achieved in the adjustment is noticeable in colorimetric terms, reaching lower magnitude residuals (Table 1) and $\Delta E_{ab}^{*}$ values (Table 2), which means better model fits and higher predictive characteristics.

#### 3.1.3. Analysis of Color Chart Patches

The use of the LOOCV procedure in this study is twofold: it allows an overall model checking as well as analyzing patches used in the camera characterization at an individual level. Values for $\Delta E_{ab}^{*}$ less than 4 CIELAB units (JND value represented by the red line in the plots in Figure 4 and Figure 5) are achieved for the majority of the patches. Also, it is clearly observed that the Fujifilm IS PRO image gives better results than the Sigma SD15 image, particularly after applying the GP (cf. Figure 5b with Figure 4b).

Table 3 displays the percentage of patches with a LOOCV $\Delta E_{ab}^{*}$ greater than 4 CIELAB units for the different regression models performed. Once again, the GP model gives slightly better results than the second-order polynomial model, especially for the Fujifilm IS PRO digital camera.

**Figure 6:**
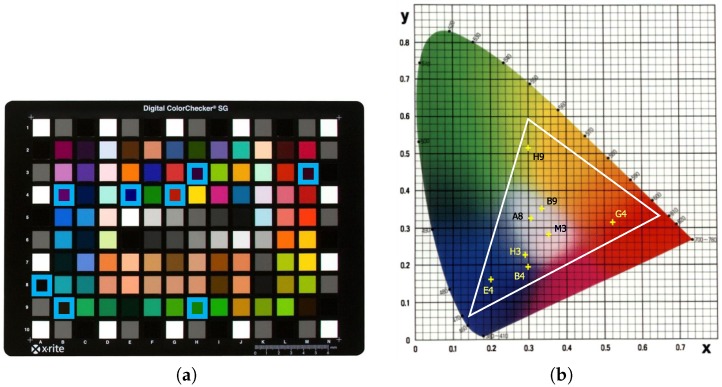
Patches with higher LOOCV $\Delta E_{ab}^{*}$ values: (**a**) on X-Rite ColorChecker; (**b**) on CIE chromaticity diagram.

Particularly, there are eight patches with the highest $\Delta E_{ab}^{*}$ values in both images regardless of the model applied (A8, B4, B9, E4, G4, H3, H9, and M3). These patches can be easily identified on the X-rite ColorChecker (Figure 6a) as well as on the CIE chromaticity diagram (Figure 6b). The worst results are found in patches E4, H9, B4, and G4 (blue, green, purple, and red, respectively). Note that patches E4 (blue), G4 (red), and H9 (green) are near the vertices of the triangle that delimits the color gamut, that is the chromatic domain, of the sRGB color space (white line plotted in picture b in Figure 6).

Thus, the nature and colorimetric characteristics of the color patches used as training sample set have an effect on the overall accuracy of the model used [58]. The colors represented with the highest $\Delta E_{ab}^{*}$ values in these patches correspond to saturated colors that are commonly found in artificial or industrial objects, but not in natural scenes such as those found in archaeological applications. We have to keep in mind that, usually, color charts used as colorimetric reference are designed mainly for industrial processes or photographic applications. Therefore, purple (B4, H3, and M3), blue (E4), green (H9 and B9), and bright red (G4) colors will not likely be present in archaeological scenes. Consequently, these color patches could be removed from the training sample during the characterization process without affecting the global accuraccy.

Previous research show that a proper selection of the patches, such as the skin tone colors, provides suitable results for camera characterization procedure applied in rock art paintings [59,60]. In Spanish Levantine rock art paintings, it is more frequent to find reddish or black colors (only dark reds in the Cova dels Cavalls) in pigments and skin tone or brown colors in the support. It is clearly observed that these patches work correctly regardless of the regression model used.

#### 3.1.4. Induced Noise Results

The two cameras used in this study have different built-in sensors. The Sigma SD15 camera incorporates a Foveon^®^X3 sensor, whereas the Fujifilm IS PRO carries a Super CCD sensor. The values of the variation coefficients were computed and compared between the input RGB image and the output sRGB characterized image for the two mathematical transformations. The pixel variability evaluation was conducted in a reduced group of ColorChecker patches with homogeneous reflectance (C7, D7, C8, and D8).

**Figure 7:**
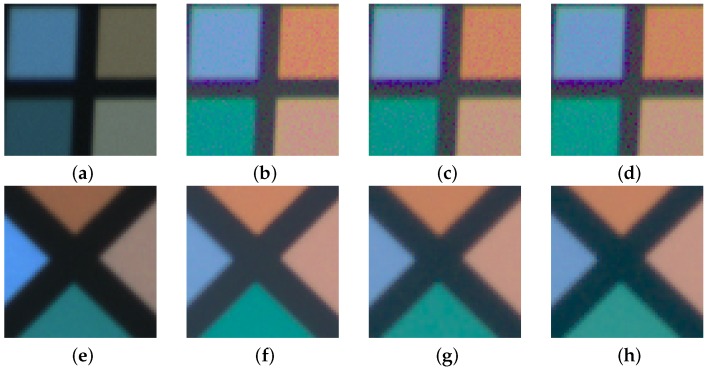
Noise comparative detail sRGB images after characterization: (**a**–**d**) Sigma SD15; (**e**–**h**) FujifilmIS PRO; (**a**,**e**) Original raw images; (**b**,**f**) Gaussian process; (**c**,**g**) Second-order polynomial model; (**d**,**h**) Linear model.

Table 4 shows the variation coefficients for the raw RGB digital values from the original images before the camera characterization (Figure 7a,e). It is informative to contrast these values with the variation coefficients obtained for the CIE XYZ (Table 5) and sRGB transformed data (Table 6) respectively. For a brief overview, Table 7 shows a summary of the variation coefficients obtained.

Moreover, as the degree of the polynomial model used can affect the results achieved in terms of noise, we included the comparison of the variation coefficients for the linear model as well [61]. Our outcomes show basically the same results in the second-order and linear models, which are still slightly worse than the GP result (cf. Table 5 and Table 6). The trend found in the induced noise results, that is, the noise depends on the sensor and therefore it is different for each camera regardless of the mathematical model.

The coefficients obtained for the digital values on the original image indicate that the noise generated directly by the Foveon^®^X3 sensor is greater than noise in the SuperCCD (Table 4). Apparently, the SuperCCD sensor seems to respond better in the raw data collection stage in terms of noise variability.

Also, the overall results confirm the superior behavior of the SuperCCD when compared with the Foveon^®^X3 sensor (cf. Table 5 and Table 6). Table 7 shows that none of the regression models applied to the Fujifilm IS PRO image increased the original variability coefficients. In fact, the coefficients decrease slightly after applying the GP; with the opposite behavior present in the Foveon^®^X3 sensor. Both, the CIE XYZ and sRGB coefficients obtained increase significantly in the Foveon^®^X3 sensor, regardless of the characterization model used.

Additionally, Figure 7 displays the noise comparative images as a result of the different color space transforms during the camera characterization. Greater noise is produced by the Foveon^®^X3 sensor versus the SuperCCD sensor. It is evident that the best results are obtained for the Fujifilm IS PRO camera in terms of noise (cf. Figure 7b–d,f–h). Obviously, the architecture of each sensor is different, as well as its characteristics and operation. Therefore, with regard to image noise, the effect produced by the sensor differs depending on the camera used. It turns out that the digital camera selected for the photographic work is a crucial aspect to be taken into account in archaeological applications.

### 3.2. Output sRGB Characterized Images

**Figure 8:**
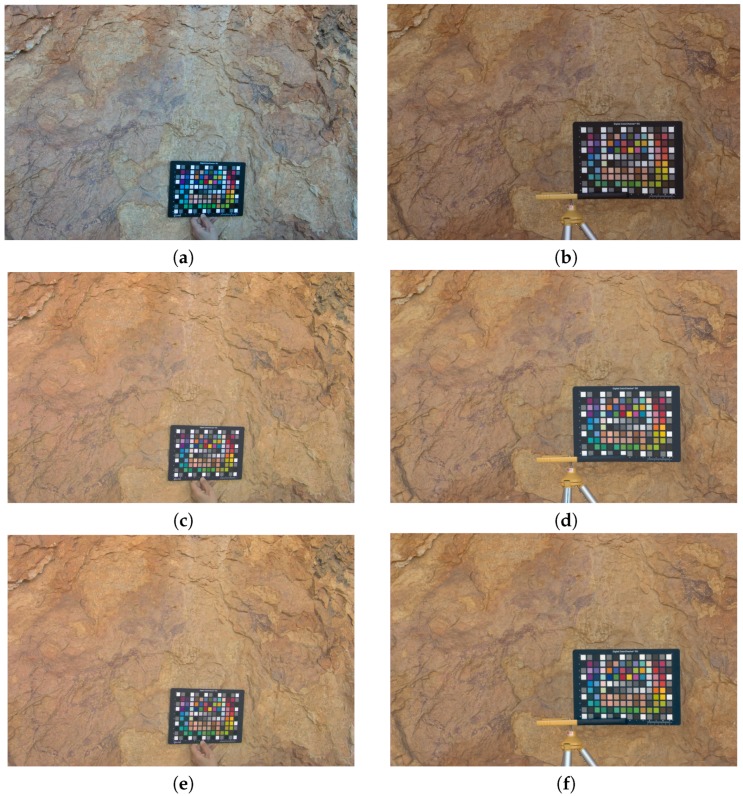
Original and output sRGB characterized images: (**a**,**c**,**e**) Sigma SD15; (**b**,**d**,**f**) Fujifilm IS PRO; (**a**,**b**) Original; (**c**,**d**) GP; (**e**,**f**) Second-order polynomial model.

The original raw images were successfully transformed into the same device-independent color space by means of the two regression models applied (Figure 8). Both the GP and the second-order polynomial model gave similar results. Even an experienced observer is unable to perceive differences between the images generated with the two regression models applied for both SLR digital cameras (cf. Figure 8c–f). The JND threshold of 4 CIELAB units is suitable in most practical applications (specifically in this study), and proves that human vision cannot perceive the improvement obtained with the GP (cf. $\Delta E_{ab}^{*}$ GP and second-order polynomial mean values in Table 2).

### 3.3. $\Delta E_{ab}^{*}$ Mapping Images

**Figure 9:**
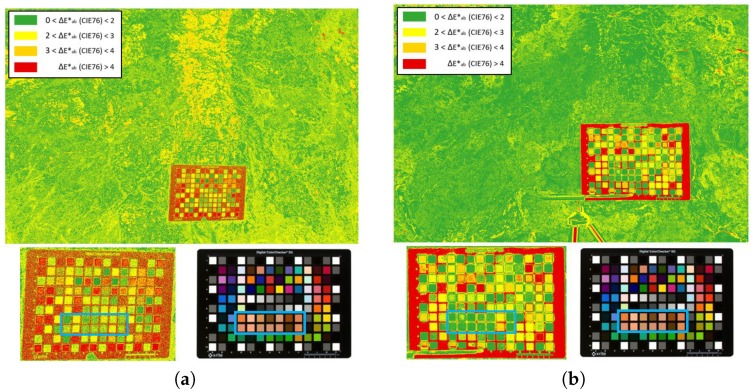
$\Delta E_{ab}^{*}$ difference mapping images between GP and second-order polynomial characterization models: (**a**) Sigma SD15; (**b**) Fujifilm IS PRO.

To verify the colorimetric quality achieved using both the GP and the polynomial regression model, we mapped the $\Delta E_{ab}^{*}$ between the two characterized sRGB images obtained (Figure 9). The predominant green color ($\Delta E_{ab}^{*}<2$ units) observed in the mapping images shows that the results obtained are very satisfactory regardless the model applied. Again, the best results were obtained for the Fujifilm IS PRO image (Figure 9b). Nevertheless, for common applications, both regression models can be used since they offer successful results.

The detail of the color chart shown in Figure 9 displays some patches marked in red, that is, with $\Delta E_{ab}^{*}$ color differences values greater than 4 CIELAB units (JND). The red color is also found on the edge of the ColorChecker. Indeed, it was on the color chart background support where we found most of the red pixels. It is well known that color depends on the incident lightin; thus, changes in geometry produce local changes of illumination in some parts of the scene. This means that in certain areas the initial homogeneous lighting hypothesis is not fulfilled, and the regression model does not fit well to the input data in shaded areas. This circumstance also reflects the importance of illumination in colorimetry.

### 3.4. Rock Art Specimen Detail Images

**Figure 10:**
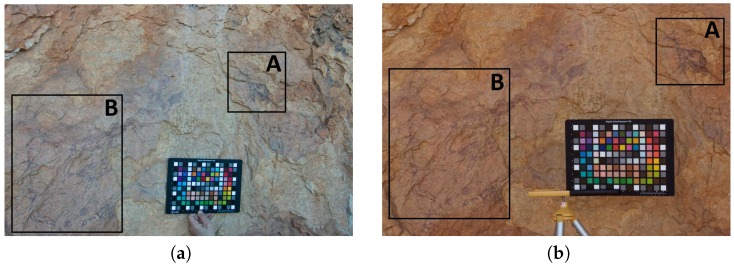
Selected rock art scenes: (**a**) SigmaSD15; (**b**) Fujifilm IS PRO. (**A**) Wounded animal detail. (**B**) Hunting scene.

**Figure 11:**
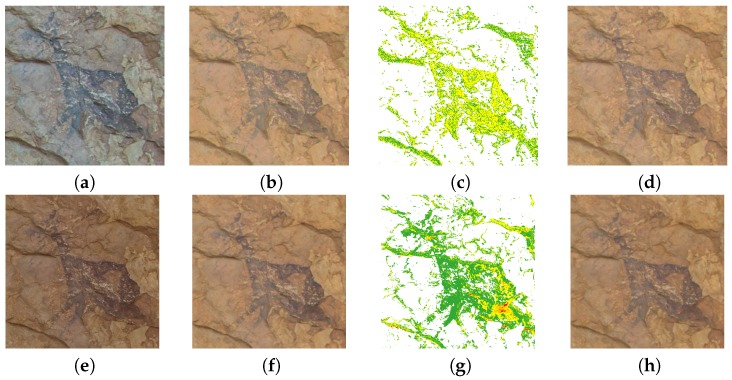
Wounded animal images: (**a**–**d**) Sigma SD15; (**e**–**h**) Fujifilm IS PRO; (**a**,**e**) Original image; (**b**,**f**) GP characterized image; (**c**,**g**) $\Delta E_{ab}^{*}$ comparative image; (**d**,**h**) Second-order characterized image.

**Figure 12:**
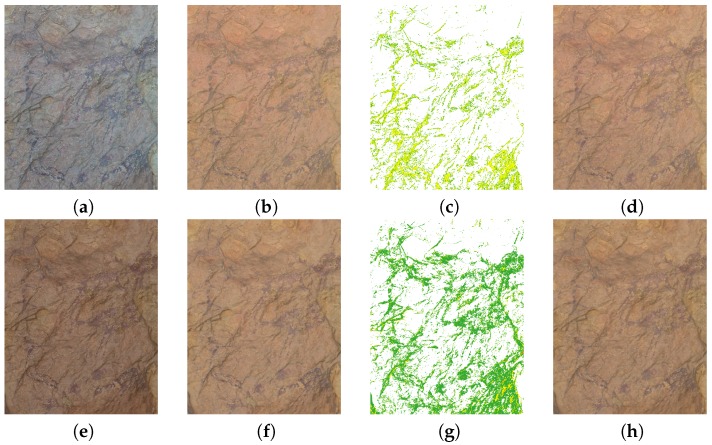
Hunter scene images: (**a**–**d**) Sigma SD15; (**e**–**h**) Fujifilm IS PRO; (**a**,**e**) Original image; (**b**,**f**) GP characterized image; (**c**,**g**) $\Delta E_{ab}^{*}$ comparative image; (**d**,**h**) Second-order characterized image.

We also compared the results obtained in two different rock art details present on the scene: the wounded animal detail (right upper corner); and the hunting scene (lower left corner) (Figure 10). For each detail selected from the scene we show the clip of the original image, the output (characterized) image after applying the different regression models, as well as the color difference mappings between both models. In order to facilitate the identification of the specimens, a mask has been applied to the latter image (Figure 11 and Figure 12).

Better results are observed in the images characterized with the Fujifilm IS PRO. The color differences $\Delta E_{ab}^{*}$ obtained for the pigments were under 2 CIELAB units, hence the predominant green color (Figure 11g and Figure 12g). A limited set of pixels marked in red ($\Delta E_{ab}^{*}>4$ CIELAB units) are observed in areas where the homogeneous lighting hypothesis is not fulfilled due to geometry changes in the support (Figure 11g). On the other hand, the yellow values ($\Delta E_{ab}^{*}$ ~ 2–3 units) present in the Sigma SD15 image can be due to the fact that the GP slightly improves the camera characterization (Figure 11c and Figure 12c). Therefore, it is important to previously make a correct selection of the camera to be used in the characterization process for archaeological research.

It can be seen that the images have been successfully characterized, independently of the regression model used. All details in the characterized images present the same ranges of colors, as well as homogeneous lighting for both cameras (cf. Figure 11b,d,f,h; Figure 12b,d,f,h).

## 4. Conclusions

The use of digital images to support cultural heritage documentation techniques has undergone unprecedented advance in the last decades. However, the original RGB data provided by digital cameras cannot be used for rigorous color measurement and communication. To face the lack of colorimetric rigor of the input RGB data recorded by the sensor, it is necessary to conduct a colorimetric camera characterization; alternatively, color profiles can also be used.

In this paper, the experimental assessment of a GP model has been carried out, and compared with a common second-order polynomial model. Although both regression models yielded good results, the use of the GP provides an improvement in colorimetric terms and fits better to the original raw RGB data. The lowest CIE XYZ residual values achieved for the adjustment and $\Delta E_{ab}^{*}$ color differences supports the use of a GP as a proper model for characterizing digital cameras. However, for practical purposes, the final sRGB characterized images derived from both the GP and the second-order polynomial model can be used with success in cultural heritage documentation and preservation tasks.

Additionally, the GP regression model has been tested on two SLR digital cameras with different built-in sensors to analyze the performance of the model in terms of pixel variability. The noise errors achieved show that similar results were obtained regardless the regression model used. However, the results also reveal that the induced noise highly depends on the camera sensor, which is clearly significant in the Foveon^®^X3 but not in the Super CCD. Thus, the correct choice of the digital camera is a key factor to be taken into consideration in the camera characterization procedure.

It is observed that the camera characterization procedure allows clear identification of the different pigments used in the scene, a proper separation from the support, the achievement of more accurate digital tracings, and accurate color measurement for monitoring aging effects on pigments. This methodology proves to be highly applicable not only in cultural heritage documentation tasks, but in any scientific and industrial discipline where a correct registration of the color is required.

## Figures and Tables

**Table 1:** Fitted CIE XYZ (Res) and LOOCV residuals (RL) values after the characterization.

	**Sigma SD15 Image**											
	**Gaussian Process**						**Second-Order Polynomial**					
	CIE X		CIE Y		CIE Z		CIE X		CIE Y		CIE Z	
	Res	RL	Res	RL	Res	RL	Res	RL	Res	RL	Res	RL
Max.	1.88	4.53	1.80	4.38	1.28	3.66	7.02	7.36	6.87	7.18	4.80	5.09
Min.	−2.48	−4.59	−3.21	−5.80	−1.50	−4.54	−4.97	−5.46	−4.70	−5.15	−3.59	−3.93
Std. Dev.	0.73	1.39	0.77	1.48	0.49	1.01	1.77	1.92	1.82	1.98	1.29	1.38
	**Fujifilm IS PRO Image**											
	**Gaussian Process**						**Second-Order Polynomial**					
	CIE X		CIE Y		CIE Z		CIE X		CIE Y		CIE Z	
	Res	RL	Res	RL	Res	RL	Res	RL	Res	RL	Res	RL
Max.	2.20	4.42	1.98	3.71	1.54	3.33	7.08	7.53	6.48	6.90	3.15	3.35
Min.	−3.72	−4.25	−2.63	−3.84	−1.35	−1.80	−4.25	−4.69	−3.12	−3.31	−1.96	−2.45
Std. Dev.	0.94	1.46	0.78	1.20	0.45	0.74	1.75	1.92	1.45	1.58	0.49	0.86

**Table 2:** $\Delta E_{ab}^{*}$ summary of the statistical results after the characterization.

	Sigma SD15 Image				Fujifilm IS PRO Image			
	Gaussian Process		Second-Order Polynomial		Gaussian Process		Second-Order Polynomial	
	$\Delta E_{ab}^{*}$	LOOCV	$\Delta E_{ab}^{*}$	LOOCV	$\Delta E_{ab}^{*}$	LOOCV	$\Delta E_{ab}^{*}$	LOOCV
Max.	8.251	8.906	15.086	17.814	8.213	8.409	12.634	13.021
Mean	1.755	2.440	2.561	2.751	1.817	2.285	2.753	2.958
Median	1.403	2.180	2.135	2.205	1.486	1.913	2.101	2.281
Std. Dev.	1.479	1.863	1.836	2.022	1.457	1.729	2.186	2.305

**Table 3:** Percentage of patches with LOOCV $\Delta E_{ab}^{*}>4$ CIELAB units.

		Sigma SD15		Fujifilm IS PRO	
		Patches	%	Patches	%
**Model**	Gaussian process	12	8.57	11	7.86
	Second-order	19	13.57	31	22.14
**LOOCV**	Gaussian process	23	16.43	22	15.71
	Second-order	20	14.29	31	22.14

**Table 4:** Noise variation coefficients of the original RGB digital numbers.

	Sigma SD15 Image			Fujifilm IS PRO Image		
Patch	R	G	B	R	G	B
C7	0.020488	0.014114	0.009923	0.011586	0.01077	0.01223
C8	0.036863	0.019129	0.017545	0.013394	0.01170	0.00851
D7	0.018434	0.016139	0.016432	0.008498	0.01294	0.01039
D8	0.015118	0.014687	0.015908	0.010909	0.01162	0.00933

**Table 5:** Variation coefficients of the output CIE XYZ coordinates.

	**Sigma SD15 Image**								
	**Gaussian Process**			**Second-Order Polynomial**			**Linear**		
**Patch**	**X**	**Y**	**Z**	**X**	**Y**	**Z**	**X**	**Y**	**Z**
C7	0.03673	0.01413	0.03559	0.02447	0.02620	0.03129	0.01988	0.04851	0.03192
C8	0.01290	0.06014	0.09700	0.01360	0.05988	0.09379	0.01916	0.04617	0.10635
D7	0.05272	0.05006	0.05985	0.07844	0.03741	0.06928	0.03755	0.05664	0.06164
D8	0.01933	0.04773	0.08251	0.01365	0.05254	0.07137	0.01632	0.04362	0.07257
	**Fujifilm IS PRO Image**								
	**Gaussian Process**			**Second-Order Polynomial**			**Linear**		
**Patch**	**X**	**Y**	**Z**	**X**	**Y**	**Z**	**X**	**Y**	**Z**
C7	0.00824	0.01098	0.00967	0.00905	0.01275	0.01009	0.01037	0.01197	0.00969
C8	0.01054	0.01162	0.01730	0.01104	0.01179	0.01782	0.00983	0.01078	0.01831
D7	0.01058	0.01286	0.01040	0.00929	0.01163	0.01066	0.00812	0.01156	0.00994
D8	0.00552	0.01033	0.01639	0.00653	0.01080	0.01632	0.00618	0.00916	0.01390

**Table 6:** Variation coefficients of the output sRGB digital numbers.

	**Sigma SD15 Image**								
	**Gaussian Process**			**Second-Order Polynomial**			**Linear**		
**Patch**	**sR**	**sG**	**sB**	**sR**	**sG**	**sB**	**sR**	**sG**	**sB**
C7	0.09597	0.02311	0.01805	0.01602	0.02768	0.07291	0.01751	0.03312	0.07113
C8	0.02104	0.06967	0.07489	0.07404	0.06745	0.01955	0.08095	0.05876	0.02398
D7	0.01982	0.03815	0.03506	0.03982	0.03447	0.04274	0.03732	0.03072	0.03744
D8	0.03110	0.05567	0.05300	0.04821	0.05360	0.02538	0.04778	0.04715	0.02661
	**Fujifilm IS PRO Image**								
	**Gaussian Process**			**Second-Order Polynomial**			**Linear**		
**Patch**	**sR**	**sG**	**sB**	**sR**	**sG**	**sB**	**sR**	**sG**	**sB**
C7	0.01693	0.00721	0.00478	0.00528	0.00825	0.01490	0.00782	0.00954	0.01624
C8	0.00712	0.00884	0.01200	0.01286	0.00922	0.00737	0.01034	0.00803	0.00671
D7	0.00462	0.00746	0.00585	0.00588	0.00748	0.00531	0.00969	0.00632	0.00577
D8	0.00163	0.00767	0.00927	0.00967	0.00776	0.00257	0.00613	0.00696	0.00324

**Table 7:** Summary of the variation coefficients obtained.

		Sigma	Fujifilm
		SD15 Image	IS PRO Image
**Original**	RGB	0.01790	0.01099
**Gaussian process**	CIE XYZ	0.04739	0.01120
	sRGB	0.04463	0.00778
**Second-order**	CIE XYZ	0.04766	0.01148
	sRGB	0.04349	0.00810
**Linear**	CIE XYZ	0.04774	0.01082
	sRGB	0.04271	0.00807

## Abbreviations

The following abbreviations are used in this manuscript:

CIE	Commission Internationale de l’Éclairage
GP	Gaussian process
LOOCV	Leave-one-out cross-validation
MCMC	Markov chain Monte Carlo
Res	Fitted CIE XYZ residuals
RL	CIE XYZ LOOCV residuals
SLR	Single Lens Reflex
